# The Signal Characteristics of Oil and Gas Pipeline Leakage Detection Based on Magneto-Mechanical Effects

**DOI:** 10.3390/s23041857

**Published:** 2023-02-07

**Authors:** Bin Liu, Qian Ge, Zihan Wu, Zheng Lian, Lijian Yang, Hao Geng

**Affiliations:** College of Information Science and Engineering, Shenyang University of Technology, Shenyang 110870, China

**Keywords:** magnetic flux leakage detection, magnetic charge model, force-magnetic coupling, magnetic flux leakage signal

## Abstract

In order to solve the problem of the quantification of detection signals in the magnetic flux leakage (MFL) of defective in-service oil and gas pipelines, a non-uniform magnetic charge model was established based on magnetic effects. The distribution patterns of magnetic charges under different stresses were analyzed. The influences of the elastic load and plastic deformation on the characteristic values of MFL signals were quantitatively assessed. The experimental results showed that the magnetic charge density was large at the edges of the defect and small at the center, and approximately decreased linearly with increasing stress. The eigenvalues of the axial and radial components of the MFL signals were compared, and it was found that the eigenvalues of the radial component exhibited a larger decline rate and were more sensitive to stress. With the increase in the plastic deformation, the characteristic values of the MFL signals initially decreased and then increased, and there was an inflection point. The location of the inflection point was associated with the magnetostriction coefficient. Compared with the uniform magnetic charge model, the accuracy of the axial and radial components of the MFL signals in the elastic stage of the improved magnetic charge model rose by 17% and 16%, respectively. The accuracy of the axial and radial components of the MFL signals were elevated by 9.15% and 9%, respectively, in the plastic stage.

## 1. Introduction

With the rapid development of the global economy and the improvement of people’s living standards, the demand for different energy resources, such as oil and natural gas, is gradually increasing. Due to the advantages of the cheap and convenient transportation of long-distance oil and gas pipelines, they are widely used in the oil and gas industries [1,2]. Long-distance oil and gas pipelines in long-term service are prone to accidents due to aging, corrosion, and other risk factors, which pose a serious threat to the global economy and people’s safety. Therefore, the safety of pipelines cannot be overlooked [3]. At present, of the majority of in-service pipelines in China are at the aging accident stage. In order to ensure the safe operation of oil and gas pipelines, they must be tested and monitored regularly [4,5]. The in-pipeline testing technology is the main nondestructive testing method, which has been developed for several decades, mainly including piezoelectric ultrasonic testing, electromagnetic ultrasonic testing, magnetic flux leakage (MFL) testing, etc. [6,7].

The MFL internal detection technology has shown an outstanding diagnostic performance [8,9], which can detect crack defects, weld defects, corrosion pits, metal losses, and other abnormal operating conditions of pipelines [10]. Compared with other detection technologies, the MFL internal detection technology possesses the advantages of non-pollution, non-coupling, high speed, and high reliability [11]. It is more appropriate for the rapid online detection of long-haul oil and gas pipelines, and it is globally recognized as the most effective detection method for the safety detection of pipelines [12,13]. The quantization of the pipeline MFL signal is the ultimate goal of MFL detection [14]. Most of the quantization methods are to carry out a large number of calibrations of experimental data [15,16], but the failure to take into account the complex situation in the pipeline will lead to poor accuracy and impracticality of the quantization method; therefore, the quantization of the theoretical model has become a hot research topic [17,18]. Under the influence of the internal pressure of the medium and the surrounding environment, there is a large stress concentration area at the defect [19,20]. The existing quantization model does not consider the influence of the stress concentration area on the magnetic leakage signal [21,22], resulting in poor quantization accuracy. Therefore, it is of great significance for the establishment of a quantitative model of magnetic leakage internal detection and quantitative calculation of the magnetic leakage signal [23,24].

In the present study, the force-magnetic coupling relationship between the non-uniform magnetic charge model and the analytical model of compound magnetic charge was developed. The distribution of the magnetic charges on the side wall under different stresses was analyzed. The influences of the elastic load and plastic deformation on the MFL signals and their characteristic values were studied. Compared with the experimental data, the reliability and accuracy of the model were verified. The research could provide a scientific basis for the quantitative detection of defects in long-distance oil and gas pipelines.

## 2. Model Development

### 2.1. Classical Model

**Figure 1:**
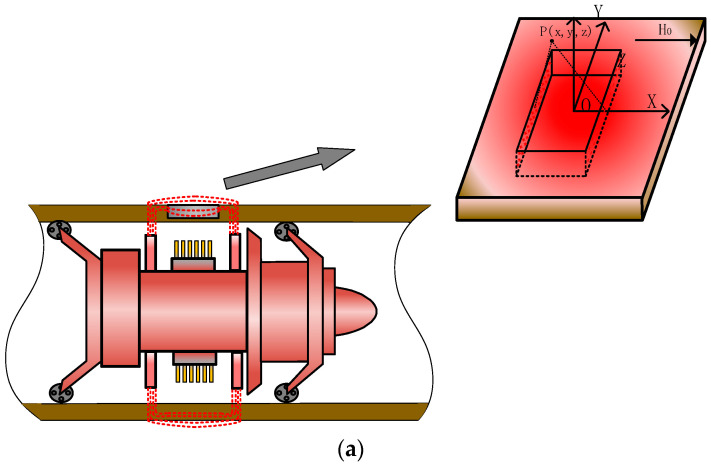

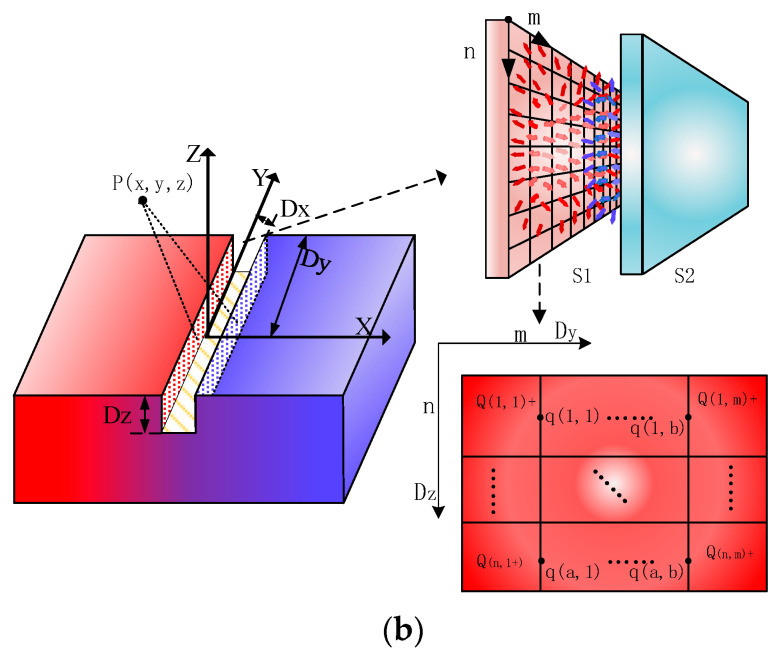
Schematic diagram of defects in pipeline and distribution of magnetic charges. (a) Schematic diagram of MFL internal detection of defects in pipeline. (b) Schematic diagram of non-uniform distribution of magnetic charges on the side wall of the defect.

The pipeline is magnetized by the excitation device of the MFL internal detection. If there is no defect in the pipeline, the magnetic lines of force pass through the inside of the pipe wall, and the probe cannot detect the magnetic leakage field in the air. When there are defects in the pipeline, the magnetic field lines in the wall will leak into the air because the permeability of the defect is small and the reluctance is large, thereby forming a magnetic leakage field, as shown in Figure 1a.

At present, magnetic charge models are widely used in the analysis of magnetic leakage fields [25,26]. As shown in Figure 1b, magnetic charges are generated at the defects and distributed on the side walls. The magnetic leakage field, when the micro-element surface dydz on the side wall is distant from point p, is formulated as follows:

(1) $$dH=\frac{\rho dydz}{4\pi \mu_{0}r^{3}}$$

where the uniform magnetic charge density is $\rho =5.3\left(\frac{D_{z}/D_{x}+1}{D_{z}/\left(D_{x}\mu \right)+1}\right)H_{0}$ [27]. The magnetic charge at the defect is affected by the shape of the defect, and the magnetic charge in the stable state presents a non-uniform distribution under the action of Coulomb force [28,29], which is difficult to overcome. As shown in Figure 1b, the rectangular defect with the defect side length dy × dz is divided into m × n sub-regions, and the side length of each sub-region is $\frac{D_{y}}{n}\times \frac{D_{z}}{m}$. Therefore, only the magnetic charge carried by each sub-region is required, and the distribution of the magnetic charge density in each sub-region can be calculated as [30]:

(2) $$\rho_{\mathrm{ij}}=\frac{Q_{\mathrm{ij}}}{s}$$

where i = 1, 2, 3... n, j = 1, 2, 3... m, p is the magnetic charge density in the subregion, Q_(i,j)_ is the magnetic charge in the subregion, and s is the area of the side wall of the defect. As illustrated in Figure 1b, the mathematical model is established in the S1 plane. The magnetic charge is repulsive in the S1 plane and attracted by the magnetic charge in the S2 plane. When the magnetic charge of the side wall of the defect is in a stable state, the vector sum of the magnetic force received by the magnetic charge is 0; thus, the Coulomb force received by the magnetic charge of any subregion is formulated as follows [31,32]:

(3) $$F=\frac{Kq_{\left(a,b\right)}Q_{\left(i,j\right)}}{r_{3}{}^{2}}+\frac{Kq_{\left(a,b\right)}Q_{\left(i,j\right)}{}_{-}}{r_{4}{}^{2}}=0$$

where Q_(i,j)+_ is the magnetic charge of any subregion in the S1 plane, Q_(i,j)-_ is the magnetic charge of any subregion in the S2 plane, and q (a, b) is the unit point magnetic charge in the plane region (a = 1, 2, 3... n − 1, b = 1, 2, 3... m − 1, a ≠ b), $r_{3}=\sqrt{{\left(\frac{D_{y}}{m}\right)}^{2}{\left(\frac{2b+1}{2}-j\right)}^{2}+{\left(\frac{D_{z}}{n}\right)}^{2}{\left(\frac{2a+1}{2}-i\right)}^{2}}$ is the distance between the magnetic charges on the same plane, $r_{4}=\sqrt{{\left(\frac{D_{y}}{m}\right)}^{2}{\left(\frac{2b+1}{2}-j\right)}^{2}+{\left(\frac{D_{z}}{n}\right)}^{2}{\left(\frac{2a+1}{2}-i\right)}^{2}+D_{x}{}^{2}}$ is the distance between the magnetic charges on different planes, and K is the Coulomb constant. Therefore, the horizontal and vertical components of any q_(a,b)_ unit magnetic charge subjected to the Coulomb force in the subregion Q_(i,j)_ are expressed as:

(4) $$F_{m}=\frac{(\frac{2b+1}{2}-j)}{r_{3}}\times \frac{Kq_{\left(a,b\right)}Q_{(i,j)+}}{r_{3}{}^{2}}+\frac{(\frac{2b+1}{2}-j)}{r_{3}}\times \frac{Kq_{\left(a,b\right)}Q_{(i,j)-}}{r_{4}}=0$$

(5) $$F_{n}=\frac{(\frac{2a+1}{2}-i)}{r_{3}}\times \frac{Kq_{\left(a,b\right)}Q_{(i,j)+}}{r_{3}{}^{2}}+\frac{(\frac{2a+1}{2}-i)}{r_{3}}\times \frac{Kq_{\left(a,b\right)}Q_{(i,j)-}}{r_{4}{}^{}}=0$$

As there are several magnetic charge regions in the plane, the force analysis of several magnetic charge regions is combined into a system of equations, as follows:

(6) $$\begin{array}{l} \left\{\begin{array}{l} \sum_{i=1}^{n}\sum_{j=1}^{m}\sum_{a=1}^{n-1}\sum_{b=1}^{m-1}F_{m}=0\\ \sum_{i=1}^{n}\sum_{j=1}^{m}\sum_{a=1}^{n-1}\sum_{b=1}^{m-1}F_{n}=0 \end{array}\right.=\\ \left\{\begin{array}{l} \left(\frac{(\frac{3}{2}-1)Kq_{{}_{\left(1,1\right)}}Q_{{}_{(1,1)+}}}{r_{3}{}^{3/2}}+\frac{(\frac{3}{2}-1)Kq_{{}_{\left(1,1\right)}}Q_{{}_{(1,1)-}}}{r_{3}\times r_{4}}+\cdots \cdots +\frac{(\frac{3}{2}-j)Kq_{{}_{\left(1,1\right)}}Q_{{}_{(i,j)+}}}{r_{3}{}^{3/2}}+\frac{(\frac{3}{2}-j)Kq_{{}_{\left(1,1\right)}}Q_{{}_{(i,j)-}}}{r_{3}\times r_{4}}=0\right)\\ \;\;\;\;\;\;\;\;\;\;\;\;\;\;\;\vdots \\ \left(\frac{(\frac{3}{2}-1)Kq_{{}_{\left(1,1\right)}}Q_{{}_{(1,1)+}}}{r_{3}{}^{3/2}}+\frac{(\frac{3}{2}-1)Kq_{{}_{\left(1,1\right)}}Q_{{}_{(1,1)-}}}{r_{3}\times r_{4}}+\cdots \cdots +\frac{(\frac{3}{2}-i)Kq_{{}_{\left(1,1\right)}}Q_{{}_{(i,j)+}}}{r_{3}{}^{3/2}}+\frac{(\frac{3}{2}-i)Kq_{{}_{\left(1,1\right)}}Q_{{}_{(i,j)-}}}{r_{3}\times r_{4}}=0\right)\\ \;\;\;\;\;\;\;\;\;\;\;\;\;\;\;\vdots \end{array}\right. \end{array}$$

Through the above-mentioned equations, the magnetic charge of Q_(n,m)+_ in any subregion can be solved. By substituting the magnetic charge in the magnetic region obtained from Equation (6) into Equation (2), the magnetic charge density expression in the S1 plane can be formulated as follows:

(7) $$\left\{\begin{array}{l} \rho_{\left(1,1\right)}=0.136\frac{D_{y}{}^{2}D_{z}{}^{2}}{D_{x}{}^{2}}\\ \rho_{\left(1,2\right)}=0.131\frac{D_{y}{}^{2}D_{z}{}^{2}}{D_{x}{}^{2}}\\ \;\;\;\;\vdots \end{array}\right.$$

**Figure 2:**
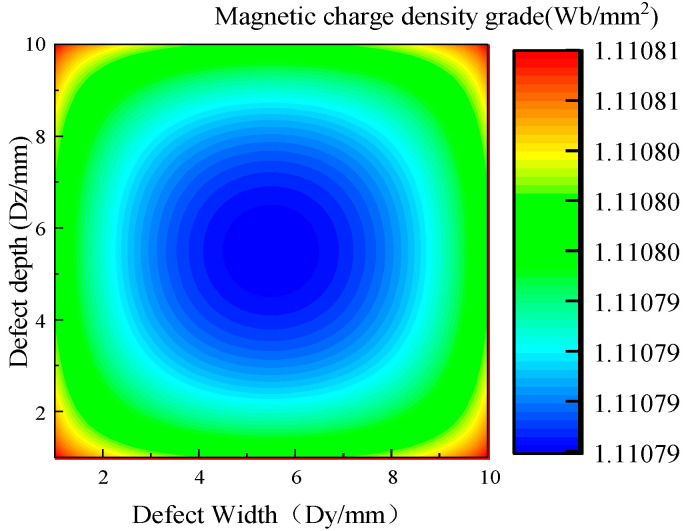
Contour map of non-uniform distribution of magnetic charges.

The non-uniform distribution of the magnetic charge on the side wall of the defect can be solved by Equation (7), as shown in Figure 2, which is the schematic diagram of the non-uniform distribution of the magnetic charge on the side wall of the S1 plane.

The magnetic charge density on the side wall of the defect presents a non-uniform distribution, and the magnetic charge density in the middle is small, whilst it gradually increases near the edge, and the magnetic charge density at the edge is the largest (Figure 2).

### 2.2. An Improved Magnetic Dipole Model

An in-service pipeline is affected by the internal pressure of the pipeline, and a stress concentration area is formed at the defect. Therefore, the establishment of the computational model of the magnetic charge density at the defect under the action of stress is more compatible with the actual condition. The magnetic field intensity at the defect can be expressed as [33]:

(8) $$H_{\mathrm{eff}}=H+H_{\sigma }^{e}+H_{\sigma }^{p}$$

where H is the strength of the excitation magnetic field, $H_{\mathrm{eff}}$ represents the effective magnetic field, $H_{\sigma }^{e}$ denotes the effective magnetoelastic field, and $H_{\sigma }^{p}$ is the effective plastic field. In the leakage magnetism, $H_{\sigma }^{e}$ can be expressed as [34]:

(9) $$H_{\sigma }^{e}=\frac{3\sigma \lambda_{s}}{\mu_{0}M_{s}^{}}\left(\cos^{2}\theta -\upsilon \sin^{2}\theta \right)$$

where θ is the angle between the direction of magnetization and the direction of stress, and v is Poisson’s ratio. In the leakage magnetism, $H_{\sigma }^{p}$ is the effective plastic field, which can be expressed as [35]:

(10) $$H_{\sigma }^{p}=\frac{6kE\varepsilon_{p}\lambda_{s}}{\mu_{0}M_{s}}-k^{'}\varepsilon_{p}^{2}M_{s}$$

Equations (9) and (10) are substituted into Equation (8) to achieve:

(11) $$H_{\mathrm{eff}}=H+\frac{3\lambda_{s}}{\mu_{0}M_{{}_{s}}^{}}\left(2kE\varepsilon_{p}+\sigma \left(\cos^{2}\theta -\upsilon \sin^{2}\theta \right)\right)-k^{'}\varepsilon_{p}^{2}M_{s}$$

According to the relationship between the magnetization of ferromagnetic materials and the magnetic charge density, it can be expressed as [36,37]:

(12) $$M=M_{s}[\coth\left(\frac{H_{eff}}{a}\right)-\frac{a}{H_{eff}}]$$

(13) $$\rho^{'}{}_{\left(i,j\right)}=\mu_{0}M_{\left(i,j\right)}=\rho_{\left(i,j\right)}[\coth\left(\frac{H_{eff}}{a}\right)-\frac{a}{H_{eff}}]$$

By substituting Equation (13) into Equation (1), the magnetic charge density under stress can be obtained. Then, the magnetic charge model under the enhanced stress can be formulated as follows:

(14) $$dH=\sum_{i=1}^{n}\sum_{j=1}^{m}\frac{\rho^{'}{}_{\left(i,j\right)}dydz}{4\pi \mu_{0}r^{3}}$$

According to the non-uniform magnetic charge model under stress, this study presented a method to quantify the MFL signals of defects [38,39].

## 3. Calculation and Analysis

In this study, the control variable method was used to assess the variations of the magnetic signal in the elastoplastic stage.

The test specimen used was a X70 pipe. The material parameter k is the ratio of elastic energy to magnetic energy, k’ is the average density of the pinpoint in the unit volume, the Young’s modulus (E) is 207 GPa, the Poisson’s ratio (υ) is 0.3, the saturation magnetization (Ms) is 1.585 × 106A/m−1, the saturation magnetostriction (λs) is 1.2 ppm, σ is the stress, the angle between the stress and magnetic field (θ) is 0°, and ε_p_ is the amount of plastic deformation.

### 3.1. Relationship between Internal Pressure and Stress

**Figure 3:**
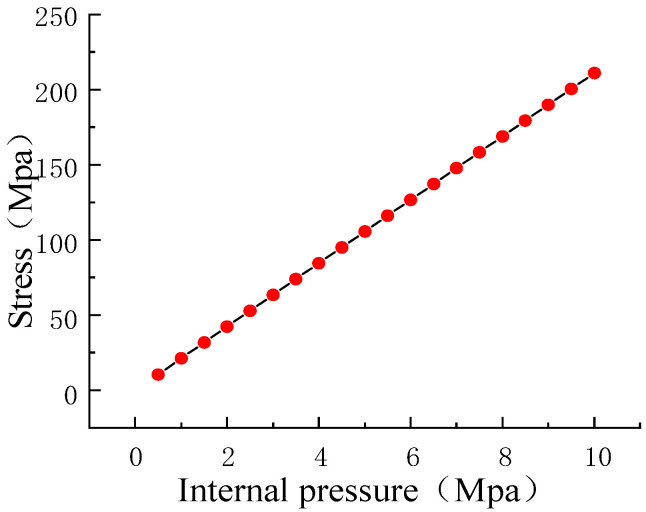
Relationship between pressure and stress in pipeline.

In the process of pipeline transportation, the internal pressure of the pipeline is within the range of 5~10 MPa, and the stress concentration area at the defect is remarkably larger than that in the other areas [40]. Therefore, the corresponding relationship between the stress at the defect and the internal pressure is formulated as follows [41,42]:

(15) $$\sigma =\frac{F\sqrt{\pi D_{y}/Q}pR}{t}$$

where *Q* is expansion coefficient, *p* is the internal pressure, *R* is the radius of pipeline, *t* is the wall thickness of the pipeline, and *D_y_* is the defect depth. In addition, *F* is the stress intensity factor at the crack tip and only depends on the crack size and internal pressure. The radius *R* of the pipelines was 259 mm, the wall thickness *t* was 12 mm, the defect depth was 10 mm, the axial length was 1 mm, and the internal pressure of 0.5~10 MPa was applied to the pipeline. The calculated stress is shown in Figure 3.

### 3.2. Effects of Stress on Magnetic Charge Density

**Figure 4:**
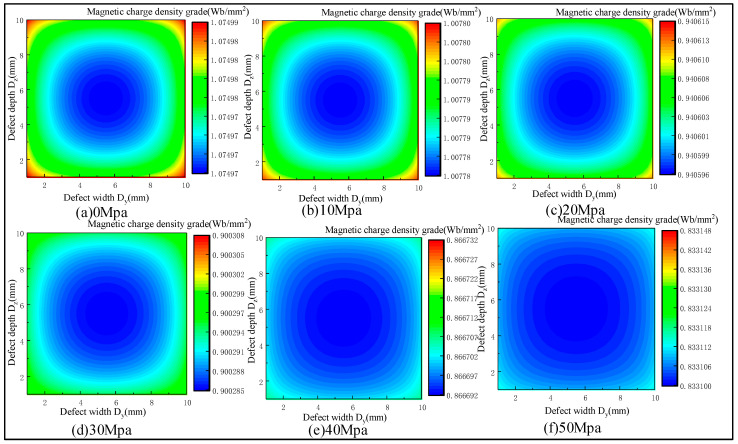
Distribution of magnetic charge density on the side wall of defects under stress.

The stress concentration area at the defect leads to the change in the magnetization of the side wall of the defect. In the present study, the defect sizes were designed with length D_x_ = 1 mm, width D_y_ = 10 mm, and depth D_z_ = 10 mm, and tensile forces of 0, 10, 20, 30, 40, and 50 MPa were applied to analyze the variation pattern of the magnetic charge density under different stresses. Figure 4 shows the distribution of the magnetic charge density on the side wall of the defects under stress.

**Figure 5:**
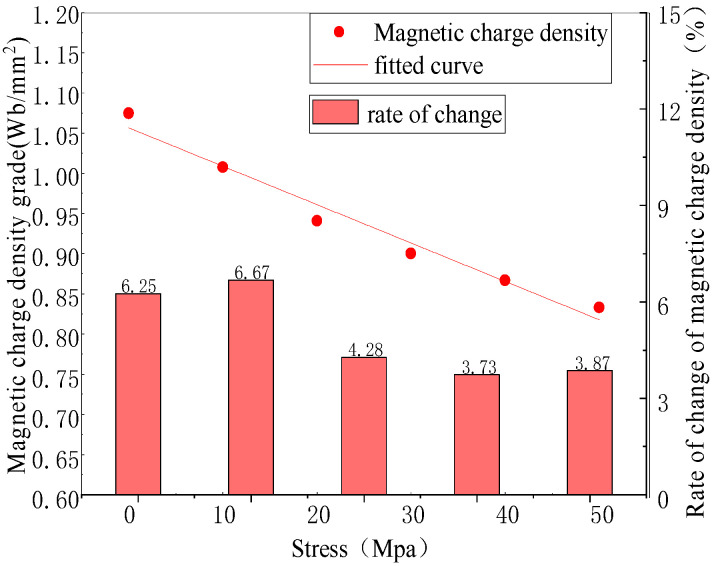
Relationship between stress and magnetic charge.

With the increase in the stress, the magnetic charge density on the side wall of the defect gradually decreased, and the overall magnetic charge presented a non-uniform distribution (Figure 4). The corresponding relationship between the stress and magnetic charge density is shown in Figure 5.

The magnetic charge density approximately decreased linearly with the increase in the stress. The rates of the change in the magnetic charge density were 6.25%, 6.67%, 4.28%, 3.73%, and 3.87%, respectively.

### 3.3. The Effects of Elastic Strain on the MFL Signals

**Figure 6:**
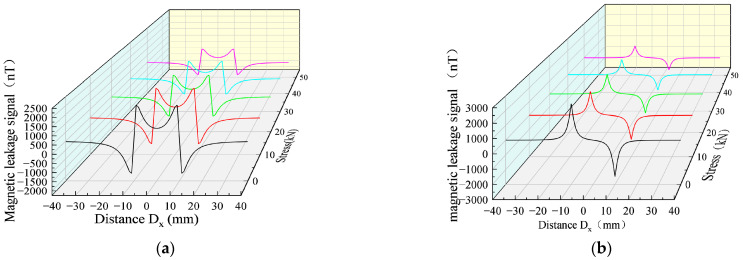
Changes of MFL signals under different stresses. (**a**) Axial components of MFL signals under different stresses. (**b**) Radial components of MFL signals under different stresses.

When the same defect size is associated with different stresses, the MFL signals are significantly different. Therefore, the MFL signals of the defect were analyzed under different stresses, the defect size was designed with length D_x_ = 10 mm, width D_y_ = 1 mm, and depth D_z_ = 1 mm, and the stresses of 0, 10, 20, 30, and 40 kN were applied (Figure 6).

**Figure 7:**
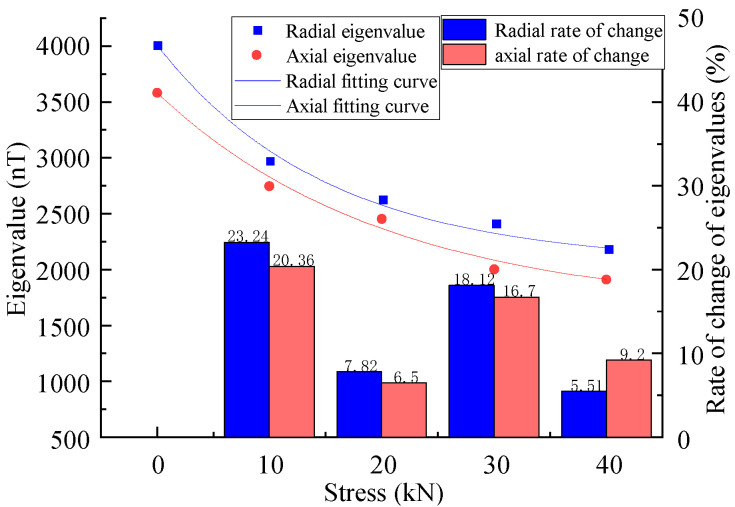
Relationship between stress and eigenvalue at the defect.

Figure 6 shows the MFL signals with defects under different stresses. It was revealed that the axial component had the maximum point and the radial component had peaks and troughs. The great eigenvalues of the axial component and the peak and valley eigenvalues of the radial component were extracted, as shown in Figure 7, for the correspondence between the stress and eigenvalue.

The results showed that the eigenvalues of the MFL signals decreased nonlinearly with the increase in the stress, where the rates of the change in the axial component eigenvalues were 20.36%, 6.5%, 16.7%, and 9.2%, and the rates of the change in the radial component were 23.24%, 7.82%, 18.12%, and 5.51%, yielding a larger rate of change in the radial component eigenvalues, which exhibited more sensitivity to stress changes (Figure 7).

### 3.4. The Influences of Plastic Deformation on MFL Signals

**Figure 8:**
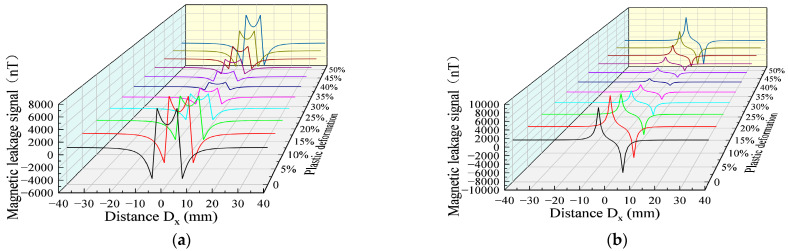
MFL signals with different plastic deformation values. (**a**) Axial component of MFL signals with different plastic deformation values. (**b**) Radial component of MFL signals with different plastic deformation values.

In the transportation process of long-distance oil and gas pipelines, the wall of the pipeline is in continuous stress under the action of long-time stress, and the plastic deformation of the defects is noteworthy. In order to study the variation trend of the MFL signals in the plastic stage, the MFL signals were analyzed under the plastic deformation values of 0%, 5%, 10%, 15%, 20%, 25%, 30%, 35%, 40%, 45%, and 50% (Figure 8).

**Figure 9:**
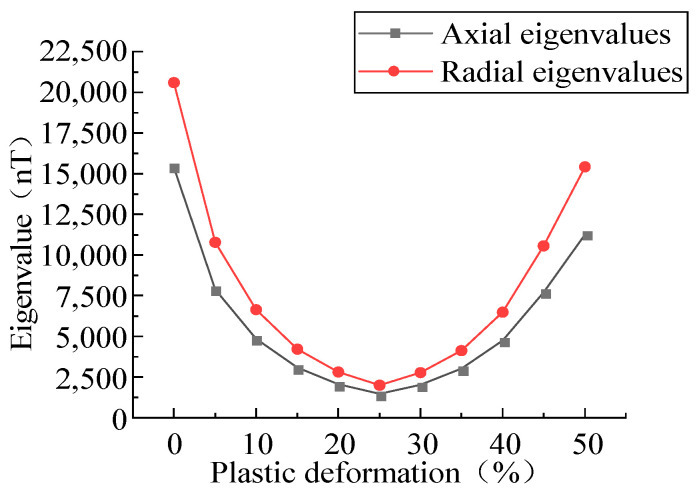
Relationship between plastic deformation and characteristic values.

With the increase in the plastic deformation, the MFL signals presented an upward trend, followed by a downward trend. The eigenvalues of the axial and radial components in the plastic deformation were extracted (Figure 9).

The MFL signals had an inflection point, a very small value, and the trend could be divided into two stages: in the first stage, a non-linear exponential reduction in the characteristics of the MLF signals was found with the increase in the plastic deformation; in the second stage, the characteristic values of the MLF signals non-linearly increased with the elevation of the plastic deformation.

**Figure 10:**
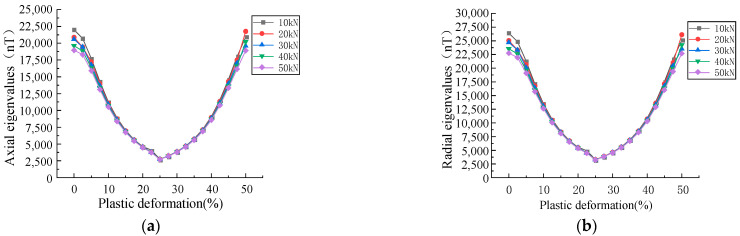
Relationship between plastic deformation and eigenvalues under different stresses. (**a**) The relationship between plastic deformation under different stresses and the eigenvalues of axial component of MFL signals. (**b**) The relationship between plastic deformation under different stresses and the eigenvalues of radial component of MFL signals.

The effects of plastic deformation on the MFL signals under different stresses and the influences of the stress on the inflection point were analyzed. As shown in Figure 10, the effects of plastic deformation on the MFL signals at stresses of 10, 20, 30, 40, and 50 kN were analyzed.

**Figure 11:**
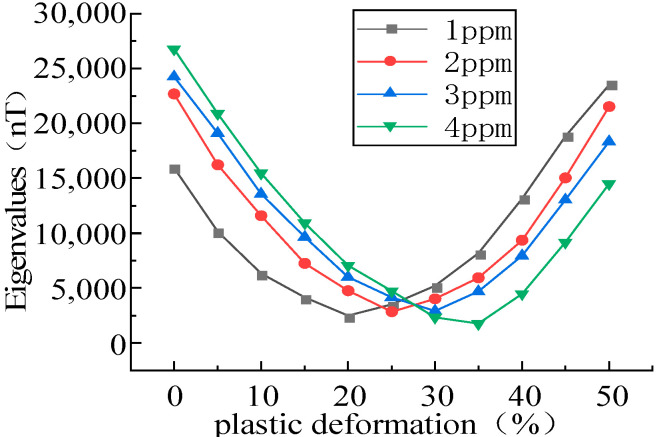
Positional relationship between magnetostriction and inflection point.

In the plastic stage, the intensity of the MFL signals decreased with the increase in the stress, initially decreased and then increased with the elevation of the plastic deformation, and an inflection point was found in the plastic deformation of 0.2–0.3 (Figure 10). As the magnetostriction coefficient λs is material-dependent, λs was analyzed and a tensile force of 10 kN was applied with magnetostriction coefficients λs of 1, 2, 3, and 4 ppm (Figure 11).

It was revealed that the magnetostriction coefficient λs affected the position of the inflection point, and with the increase in the magnetostriction coefficient λs, the inflection point shifted to the right. Therefore, it could be concluded that the inflection point was associated with the properties of ferromagnetic materials (Figure 11).

### 3.5. Comparative Analysis of Improved Magnetic Charge Model and Classical Magnetic Charge Model with Different Defect Sizes

#### 3.5.1. Comparative Analysis of Improved Magnetic Charge Model and Classical 

##### Magnetic Charge Model under Different Defect Lengths

**Figure 12:**
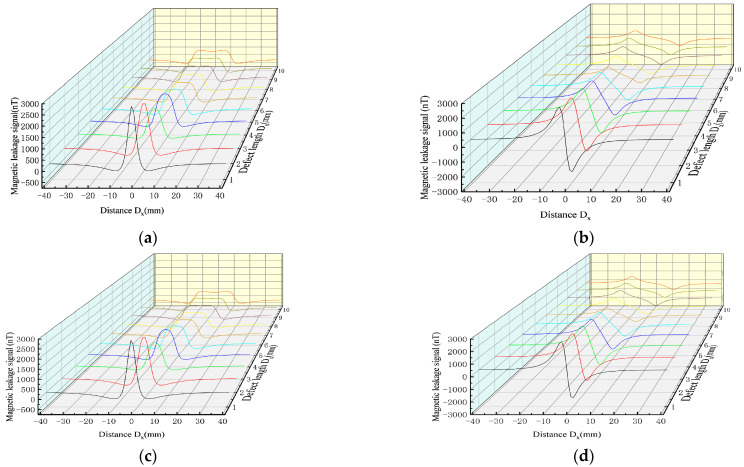
Comparative analysis of MFL signals between improved magnetic charge model and classical magnetic charge model under different defect lengths. (**a**) MFL of the axial component of improved magnetic charge model with different defect lengths. (**b**)MFL of the radial component of improved magnetic charge model with different defect lengths. (**c**) MFL of axial components of classical magnetic charge model with different defect lengths. (**d**) MFL of radial components of classical magnetic charge model with different defect lengths.

In order to study the variation rules of the magnetic leakage signals of the improved model and the classical model under different defect lengths, the width D_y_ = 1 mm, depth D_z_ = 1 mm, and length D_x_ were set as 1–10 mm to simulate the variation rules of the magnetic leakage signals with different defect lengths. (Figure 12).

**Figure 13:**
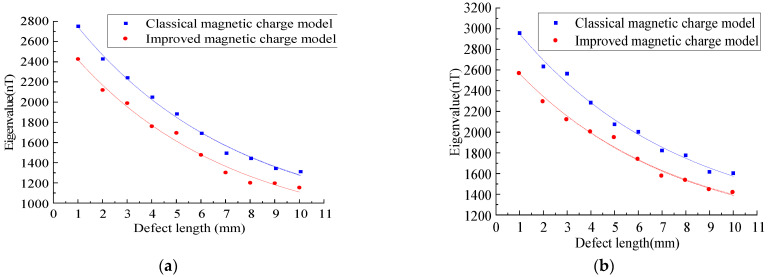
Analysis of characteristic values under different defect lengths. (**a**) Analysis of axial eigenvalues under different defect lengths. (**b**) Analysis of radial eigenvalues under different defect lengths.

As shown in Figure 12, the improved magnetic charge model and the classical magnetic charge model decrease with the increase in the defect length, the axial component has a double peak value, and the distance between the peak and valley values of the radial component becomes larger, and the change trend is consistent. As shown in Figure 13, the variation rule of the eigenvalues is studied.

As shown in Figure 13, it can be seen that with the increase in the defect length, the characteristic value of the improved model decreases nonlinearly, and the characteristic value of the improved magnetic charge model is smaller than that of the classical magnetic charge model.

#### 3.5.2. Comparative Analysis between the Improved Magnetic Charge Model and the Classical Magnetic Charge under Different Defect Depths

**Figure 14:**
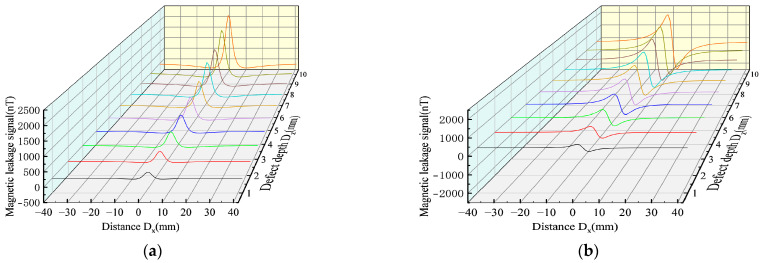

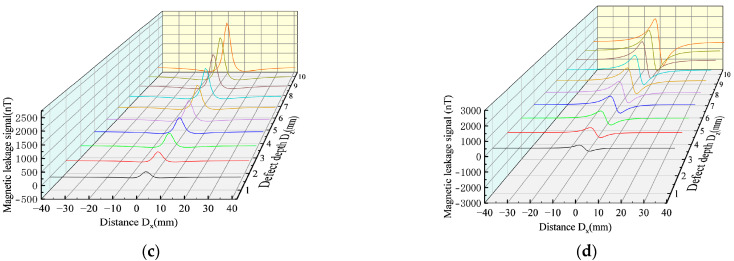
Comparative analysis of MFL signals between the improved magnetic charge model and the classical magnetic charge model under different defect depth. (**a**) MFL of the axial component of the improved magnetic charge model with different defect depth. (**b**) MFL of the radial component of the improved magnetic charge model with different defect depth. (**c**) MFL of axial components of classical magnetic charge model with different defect depth. (**d**) MFL of radial components of classical magnetic charge model with different defect depth.

As can be seen from Equation (7), the influence results of the depth and width are consistent. Therefore, the variation rules of the magnetic leakage signals of the improved model and the classical model under different defect depths were studied. The width D_y_ = 1 mm, length D_x_ = 1 mm, and depth D_z_ were set as 1–10 mm to simulate the variation rules of the magnetic leakage signals with different defect lengths. (Figure 14).

**Figure 15:**
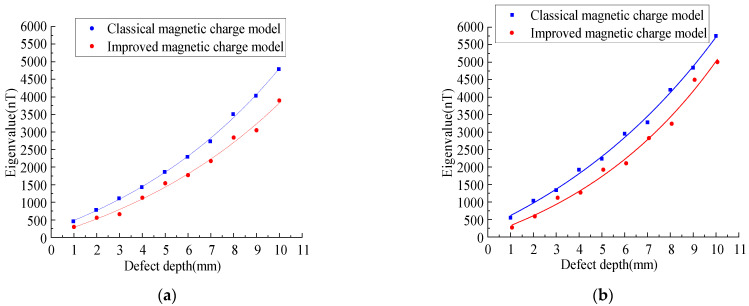
Analysis of characteristic values under different defect depth. (**a**) Analysis of axial eigenvalues under different defect depth. (**b**) Analysis of radial eigenvalues under different defect depth.

As shown in Figure 14, the MFL signals of the improved magnetic charge model and the classical magnetic charge model increase with the increase in the defect depth, and the peak-valley value spacing of the radial component does not change. Figure 15 shows the variation rules of the characteristic values under different defect depths.

As shown in Figure 15, it can be seen that with the increase in the defect depth, the eigenvalues increase nonlinearly, and the eigenvalues of the improved magnetic charge model are smaller than the classical magnetic charge model.

## 4. Experiment and Analysis

In order to verify the influences of the elastic-plastic stage on the MFL signals under different stresses, the trend of the change in the MFL signals was analyzed by tensile strip testing of an X70 steel pipe. The machining defects in the X70 steel pipe were analyzed using EDM technology. The chemical composition and mechanical properties of X70 steel are shown in Table 1.

### 4.1. Experimental Equipment and Materials

**Figure 16:**
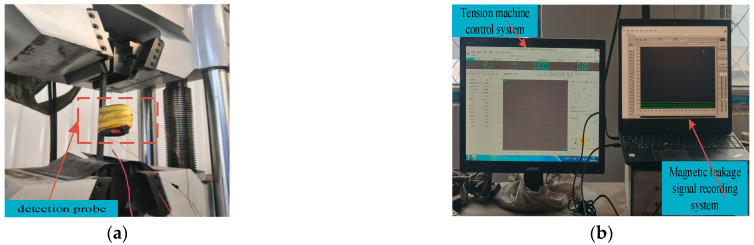
X70 steel strip tensile test device. (**a**) Tensile testing machine. (**b**) Software control systems and signal acquisition systems.

As shown in Figure 16, the experimental device was mainly composed of a tensile testing machine, X70 ferrimagnet, software control system, and signal acquisition system.

The size of the ferromagnetic material type used was 800 × 60 × 16 mm^3^, the cross-sectional area was 60 × 16 mm^2^, and the defect size was 16 × 1 × 2 mm^3^. The tensile tester model SHT-4106, with clamps on the top and bottom sides, was utilized to tighten and apply tension to the ferromagnetic materials. The probe in the signal acquisition system was a three-axis Mlx90393 magnetic induction sensor with a 16-bit magnetic field resolution, the lifting distance of which is 2 mm. (Figure 16).

### 4.2. Experimental Analysis and Verification of Elastic Deformation

**Figure 17:**
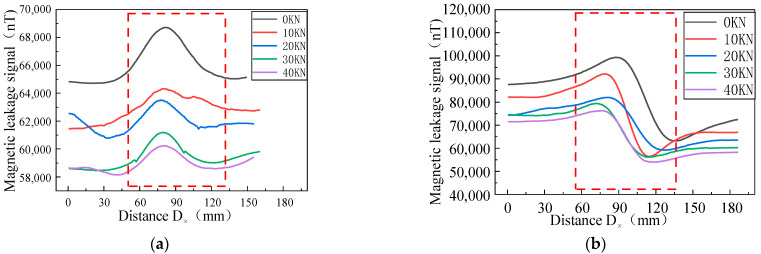
MFL signals under different stresses. (**a**) Axial component of MFL signals. (**b**) Radial component of MFL signals.

Uniform tensile forces of 0, 10, 20, 30, and 40 kN were applied to the steel pipe, and the magnitudes of the MFL signals were recorded. Figure 17 shows the MFL signals of the axial and radial components of the X70 specimen in the elastic stage under different tensile forces.

**Figure 18:**
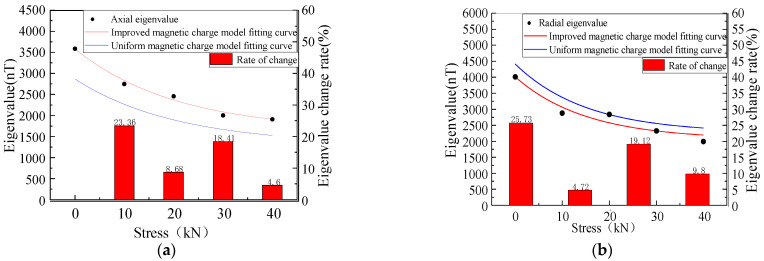
Eigenvalues under different stresses. (**a**) Axial component fitting and eigenvalue analysis. (**b**) Radial component fitting and eigenvalue analysis.

The MFL signals recorded by the experimental equipment are illustrated in Figure 17. With the increase in the pressure, the intensity of the MFL signals gradually decreased, and the peak values of the axial and radial components were gradually reduced. The peak value of the axial component and the peak value of the radial component were extracted and studied. The MFL signals under different stresses are shown in Figure 18.

It was revealed that in the elastic stage, with the increase in the tension, the characteristic values of the axial and radial components nonlinearly decreased. The variation trend of the experimental data was consistent with that of the calculated model, and the characteristic values of the radial component were larger than those of the axial component. Compared with the uniform magnetic charge model, the accuracy of the characteristic values of the axial and radial components of the modified magnetic charge model was 87% and 88%, and the accuracy of the characteristic values of the axial and radial components of the uniform magnetic charge model was 70% and 72%, which increased by 17% and 16%, respectively.

### 4.3. Experimental Analysis and Verification of Plastic Deformation

As shown in Figure 15, the plastic deformation of the ferromagnetic signal material is 10%, 20%, 30%, 40% and 50% under different pulling forces.

**Figure 19:**
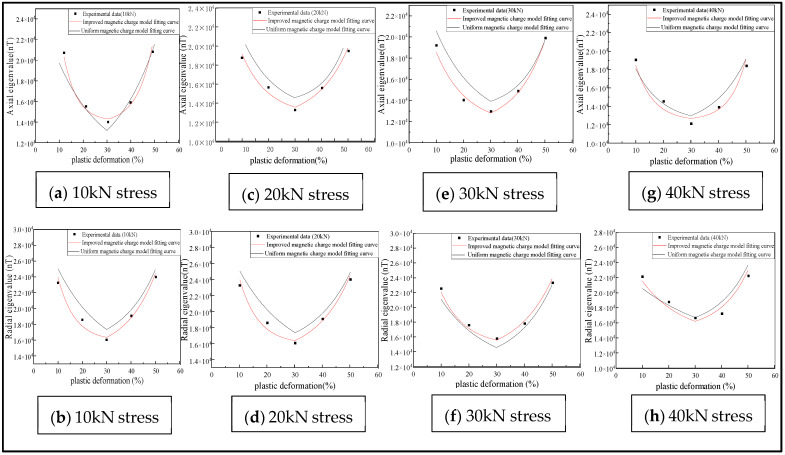
Relationship between plastic deformation and eigenvalue under different stresses.

It was revealed that the variation patterns of the characteristic values under different pulling forces were similar (Figure 19). With the increase in the pulling forces, the characteristic values of the MFL signals were reduced. With the increase in the plastic deformation, the eigenvalue initially decreased, and then increased gradually. The improved magnetic charge model and the uniform magnetic charge model were compared and analyzed (Table 2).

It can be concluded from Table 2 that the average accuracy of the eigenvalue of the axial component of the improved magnetic charge model was 84.55%, and that of the radial component was 86.1%. The average accuracy of the eigenvalues of the axial component and the radial component of the uniform magnetic charge model was 75.4% and 77.1%, which increased by 9.15% and 9%, respectively. Therefore, the model can be used to predict the variation pattern of the characteristic values of MFL signals in the plastic deformation stage under different stresses.

## 5. Conclusions

This study presented the force-magnetic coupling relationship for the non-uniform magnetic charge model, established a theoretical model of compound magnetic charge, and analyzed the variation pattern of characteristic values of MFL signals under the influences of elastic stress and plastic deformation. The research results can be summarized as follows:

(1) The characteristic values of the MFL signals under elastic stress gradually decreased with the increase in the stress, and the characteristic values of axial and radial components conformed to the nonlinear decline.
(2) At the plastic stage, the characteristic values of the MFL signals initially increased, and then decreased, with the rise of the deformation, showing an inflection point. This may be attributed to the material properties. The radial component of the MFL signals exhibited sensitivity to the change in the plastic deformation.
(3) The MFL signals in the elastic and plastic stages were compared with the uniform magnetic charge model. The accuracy of the axial component of the MFL signals in the elastic stage rose by 16%, and the accuracy of the radial component of the MFL signals was elevated by 17%. The accuracy of the axial component of the MFL signals and the radial component of the MFL signals increased by 9.15% and 9%, respectively, in the plastic stage. It can effectively predict the magnitude of MFL signals under stress.

## Figures and Tables

**Table 1:** Chemical composition and mechanical properties of X70 steel pipe.

Chemical Component (%)					Mechanical Property	
C	Si	Mn	P	S	Strength of Extension (Mpa)	Yield Strength (Mpa)
0.12	0.45	1.07	0.025	0.015	570	485–638

**Table 2:** Precision analysis of the improved magnetic charge model and uniform magnetic charge model.

	Accuracy of Improved Magnetic Charge Model (%)		Accuracy of Uniform Magnetic Charge Model (%)	
Stress (kN)	Axial Component	Radial Component	Axial Component	Radial Component
10	85.2	87.3	75.8	76.5
20	84.4	85.1	73.5	74.3
30	85.2	85.6	73.7	78.2
40	83.4	86.4	78.6	79.4

## Data Availability

Not applicable.
